# Choriocapillaris Loss in Advanced Age-Related Macular Degeneration

**DOI:** 10.1155/2018/8125267

**Published:** 2018-01-30

**Authors:** Carlos A. Moreira-Neto, Eric M. Moult, James G. Fujimoto, Nadia K. Waheed, Daniela Ferrara

**Affiliations:** ^1^Department of Ophthalmology, Tufts University School of Medicine, Boston, MA, USA; ^2^Hospital de Olhos do Paraná, Curitiba, PR, Brazil; ^3^Department of Electrical Engineering and Computer Science and Research Laboratory of Electronics, Massachusetts Institute of Technology, Cambridge, MA, USA

## Abstract

The purpose of this review is to summarize the current knowledge on choriocapillaris loss in advanced age macular degeneration (AMD). Several histopathological studies in animal models and human eyes had showed that the choriocapillaris density decreases with age. However, the role of choriocapillaris loss is still unclear in AMD and its advanced forms, either choroidal neovascularization (CNV) or geographic atrophy (GA). Some authors have hypothesized that choriocapillaris loss might precede overt retinal pigment epithelium atrophy. Others have hypothesized that deposition of complement complexes on and around the choriocapillaris could be related to the tissue loss observed in early AMD. The development of imaging modalities, such as optical coherence tomography angiography (OCTA), have led to a better understanding of underlying physiopathological mechanisms in AMD. OCTA showed atrophy of choriocapillaris underneath and beyond the region of photoreceptors and RPE loss, in agreement with previous histopathologic studies. The evolution of OCTA technology suggests that CNV seems to originate from regions of severe choriocapillaris alteration. Significant progress has been made in the understanding of development and progression of GA and CNV. *In vivo* investigation of the choriocapillaris using OCTA may lead to new insights related to underlying disease mechanisms in AMD.

## 1. Introduction

A major biological function of the choriocapillaris is to supply oxygen and metabolites to the RPE and outer neurosensory retina, constituting the only route for metabolic exchange in the retina within the foveal avascular zone. This route is also responsible for removing and recycling the wastes from the neurosensory retina [1, 2].

Aging is a complex multifactorial process that leads to ultrastructural changes to the retinal pigment epithelium (RPE), Bruch's membrane, and choriocapillaris [3]. Electron microscopy has shown that aged human Bruch's membrane has abnormalities analogous to what was observed in descriptive and experimental studies of human systemic vascular aging and atherosclerosis [4]. Normal aging changes to the choriocapillaris have been described in experimental mouse models [5, 6] as well as human eyes [7], including ultrastructural damage to the endothelial cells and choriocapillaris atrophy [3]. Interestingly, the glomerulus of kidney is a comparison organ for the RPE-Bruch's membrane complex because of their common biological functions of filtration and molecular similarities of their basement membranes. Some of these aging changes in the eye are comparable to renal tubular epithelial cell changes associated with acute interstitial nephritis and acute tubular necrosis [3, 8, 9]. Ramrattan and colleagues showed in a morphometric study of 95 unpaired normal human aging eyes that the density of the choriocapillaris decreases with age [10]. Choriocapillaris endothelial cell fenestration loss was also observed adjacent to large outer collagenous layer deposits, but not with isolated choriocapillaris basement membrane alterations, which may be a sign of cytotoxic injury in human aging eyes with age-related macular degeneration [11].

## 2. Choriocapillaris in GA

Advanced nonexudative age-related macular degeneration (AMD) is characterized by drusen, pigmentary changes, and eventual loss of photoreceptors, RPE, and choriocapillaris in a distinct geographic atrophy (GA) lesion. Although significant progress has been made in the understanding of risk factors associated with development and progression of AMD and GA, the role of choriocapillaris loss is still unclear. While RPE loss is the hallmark of GA lesions, some authors have recently hypothesized that photoreceptor loss or choriocapillaris loss might precede the overt RPE atrophy [12].

Extensive experimental and genetic evidences suggest a major role of the alternative complement pathway in the development of AMD and GA [13]. It has been hypothesized that deposition of complement pathway complexes on and around the choriocapillaris could be related to the choriocapillaris loss observed since early AMD, correlating with the abundance and size of drusen [14]. Mullins and colleagues investigated whether eyes from donors with a high-risk genotype associated with complement gene polymorphism exhibited altered levels of membrane attack complex (MAC) in the choroid, compared to eyes with a low-risk genotype. These authors showed that eyes from donors with high-risk genotype had 69% higher levels of MAC than low-risk controls, independent of any clinical signs of AMD. Their results provide evidence that high-risk complement-related genotypes may affect AMD risk by increased deposition of MAC around the aging choriocapillaris [15].

This same group evaluated the abundance of MAC in normal aging eyes, early AMD, and advanced AMD donor eyes. These authors found that samples from those with AMD had variable but significantly higher levels of MAC than either age-matched control eyes or younger eyes. Using MAC immunofluorescence, they found that in eyes with early AMD, small hard drusen were almost invariably labeled with anti-MAC antibody. In contrast to younger eyes and aged control eyes, extension of the MAC reactive domain often extended into the outer choroid. In the aging macula, MAC was predominantly localized to the outer aspect of Bruch's membrane and in extracellular domain surrounding the choriocapillaris. In eyes with GA, MAC was present in the choriocapillaris outside of areas of RPE and photoreceptor loss in a pattern similar to that seen in early AMD, although reactivity on outer vessel walls was more notable in eyes with GA. In areas of extensive atrophy, the intensity of immunoreactivity at the choriocapillaris/Bruch's membrane interface was lower than elsewhere, although a moderate level of anti-MAC labeling was found to persist even when RPE, photoreceptor, and choriocapillaris loss was complete [16].

Aiming at a better understanding of MAC accumulation in the choroid and other aging tissues, Chirco and colleagues studied the abundance of MAC across multiple human tissues. They concluded that selective accumulation of MAC in the choriocapillaris is a plausible explanation for the fact that individuals with high-risk genotypes develop AMD rather than an array of extraocular diseases. The choroid appears to be a “hot spot” for MAC deposition [17].

Zeng and colleagues describe the effects of complement exposure on choroidal endothelial cells in a system that models some aspects of AMD. Their results indicate that when choriocapillaris is exposed to MAC, choroidal endothelial cells are susceptible to complement-mediated cytolysis in a concentration- and dose-dependent manner [18].

Seddon and colleagues hypothesized, based on a histopathological study, that RPE atrophy might precede choriocapillaris loss in GA. However, they also observed that choriocapillaris loss occurred in the absence of RPE atrophy in few eyes with early AMD [19].

## 3. OCTA Documenting Choriocapillaris in GA

Optical coherence tomography (OCT) is a key imaging modality in the evaluation and management of chorioretinal diseases, allowing noninvasive optical reconstruction of the anatomy based on back-reflected light. Despite the ability of OCT to image *in vivo* structures with resolution approaching histological section, it is fundamentally limited in the detailed documentation of the microvasculature of the fundus [20].

To visualize chorioretinal vasculature without the need for intravenous dye, several OCT-based angiography technologies have been developed for a three-dimensional vascular mapping of the microcirculation [21]. OCT angiography (OCTA) is a new imaging modality that employs motion contrast imaging to high-resolution, dense volumetric datasets generating angiographic images noninvasively. OCTA computes the decorrelation signal, based on difference in the backscattered OCT signal intensity or amplitude between sequential OCT scans taken at precisely the same location, in order to generate a blood flow map [22]. OCTA requires higher imaging speeds than structural OCT because it acquires repeated B-scans at each retinal location. In addition, sophisticated algorithms are also required to manage image artifacts, ensuring that the resultant OCTA images represent strictly blood cells' movement in chorioretinal blood vessels [23, 24].

The dynamic range of OCTA is limited in commercially available devices, so there is a slowest detectable flow and a fastest distinguishable flow. Blood flowing below the slowest detectable flow produces decorrelation signals that cannot be separated from system noise and are therefore undetectable with the currently available technology. Blood flowing faster than the fastest distinguishable flow produces similar decorrelation and are therefore indistinguishable from one another [25].

Despite current limitations, OCTA provides a unique opportunity for *in vivo* assessment of the choriocapillaris. In patients with GA, OCTA with swept-source technology showed atrophy of choriocapillaris underneath the region of photoreceptor and RPE loss, in agreement with previous histopathologic studies [26, 27]. In some cases, choriocapillaris alterations on OCTA and histopathology related to impaired flow and dropout were found extending beyond the margins of GA or between discrete areas of GA [16]. In other cases, however, choriocapillaris alterations on OCTA were grossly aligned with the boundaries of the GA lesion on fundus imaging [25].

**Figure 1:**
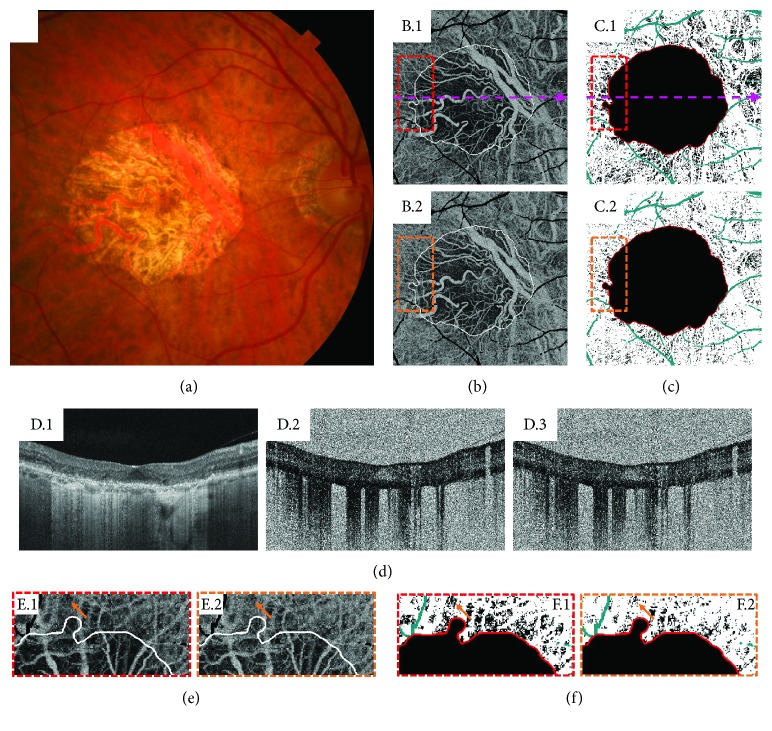
A 76-year-old patient with geographic atrophy (GA). (a) Color fundus photograph. (b) En face projections of a 6 mm × 6 mm, unthresholded optical coherence tomography (OCT) angiography (OCTA) volume from Bruch's membrane to 45 *μ*m below. B.1 corresponds to a 1.5 ms interscan time OCTA volume, and B.2 corresponds to a 3.0 ms interscan time OCTA volume. Projection artifacts from large retinal vessels have been removed and colored black. The white contours trace the margin of atrophy, as determined by a subretinal pigment epithelium (RPE) slab of the OCT volume. Note that the 1.5 ms interscan OCTA image reveals substantially more choriocapillaris alteration than does the 3.0 ms interscan time image. In some regions, the OCTA signal is documented in the 3.0 ms interscan time but not the 1.5 ms interscan time, suggesting that these regions have flow impairment rather than complete choriocapillaris atrophy. (c) Binarized versions of the choriocapillaris OCTA images in B, where a constant threshold was used. C.1 corresponds to the 1.5 ms interscan time OCTA image, and C.2 corresponds to the 3.0 ms interscan time OCTA image. Again, note there are substantially more areas of low choriocapillaris flow (black) in the 1.5 ms interscan time OCTA image than in the 3.0 ms interscan time OCTA image. (d) OCT and OCTA B-scans extracted from the locations indicated by the dashed pink lines of B.1 and C.1. The OCT B-scan (D.1) shows RPE and photoreceptor loss, which causes increased light penetration into the choroid. The 1.5 ms OCTA B-scan is shown in D.2, and the 3.0 ms OCTA B-scan is shown in D.3. Note that both D.2 and D.3 are unthresholded OCTA images, which results in worse image quality. Unthresholded choriocapillaris OCTA images are useful for reducing the rate of false-positive flow impairment due to thresholding. (e-f) Enlargements of the dashed boxes in B-C. Red boxes correspond to 1.5 ms interscan time images, and orange boxes correspond to 3.0 ms interscan time images. The boxes have been rotated 90 degrees clockwise relative to their orientations in B and C. These regions of interest show that there is choriocapillaris flow impairment beyond the margin of RPE atrophy. Arrows point to an example area of flow impairment which changes as a function of interscan time. Note that in the 1.5 ms OCTA, there is less OCTA signal (more dark areas) than in the 3.0 ms OCTA, which makes the impairment more pronounced in the 1.5 ms OCTA (this is easiest seen in F.1 and F.2). This illustrates how shorter interscan time OCTA is more sensitive to flow alterations than is longer interscan time OCTA.

Choi and colleagues used ultrahigh-speed swept-source OCTA and variable interscan time analysis (VISTA) algorithm to assess choriocapillaris changes in patients with GA. Although VISTA has the ability to shift the range downwards of the detectable flow speeds, these authors still highlighted some challenges in the interpretation of OCTA images. A low decorrelation signal may be observed due to a complete absence of flow and vasculature, secondary to true vascular atrophy. However, a low decorrelation signal may also be observed due to slow blood flow but intact vasculature, secondary to flow impairment only. Collectively, atrophy and flow impairment represent different types of choriocapillaris alteration. In this same study, OCTA with VISTA was used to study choriocapillaris flow alterations beyond the margins of GA (Figure 1); OCTA was also used to identify choroidal neovascularization (CNV) in two cases that have not been diagnosed with other imaging modalities [25].

## 4. Choriocapillaris in Neovascular AMD

Regarding the role of choriocapillaris in neovascular AMD, McLeod and colleagues analyzed three postmortem eyes correlating with ocular medical history and demographic information as available and compared them to control eyes. The percentage of RPE coverage and vascular area by choriocapillaris in the regions 1 mm outside of the CNV was 95.9% ± .8% and 39.6% ± 15.9%, respectively. The decrease in choriocapillaris vascular area was evident well beyond the submacular region and in one case extended peripherally 10 mm from the CNV into the equatorial choroid. Compared with aged control eyes, the percentage vascular area in the regions of 1 mm outside of the CNV was significantly reduced, reflecting the loss of interconnecting capillary segments in these regions. There was no significant difference in vessel diameters between the aged control eyes and the viable capillaries in neovascular AMD eyes 1 mm outside the CNV area [11]. Biesemeier and colleagues also analyzed postmortem eyes with neovascular AMD and found that the choriocapillaris was severely affected. In their opinion, the loss of choriocapillaris in neovascular AMD is counteracted by the formation and growth of new blood vessels [12]. In 2016, Seddon and colleagues speculated that hypoxic RPE resulting from reduced blood supply might upregulate production of vascular endothelial growth factor, providing the stimulus for neovascular disease [19]. According to Dryja, these findings suggest that abnormalities of the choriocapillaris may predate piercing of Bruch's membrane by months or years [28].

## 5. OCTA Documenting Choriocapillaris in Neovascular AMD

**Figure 2:**
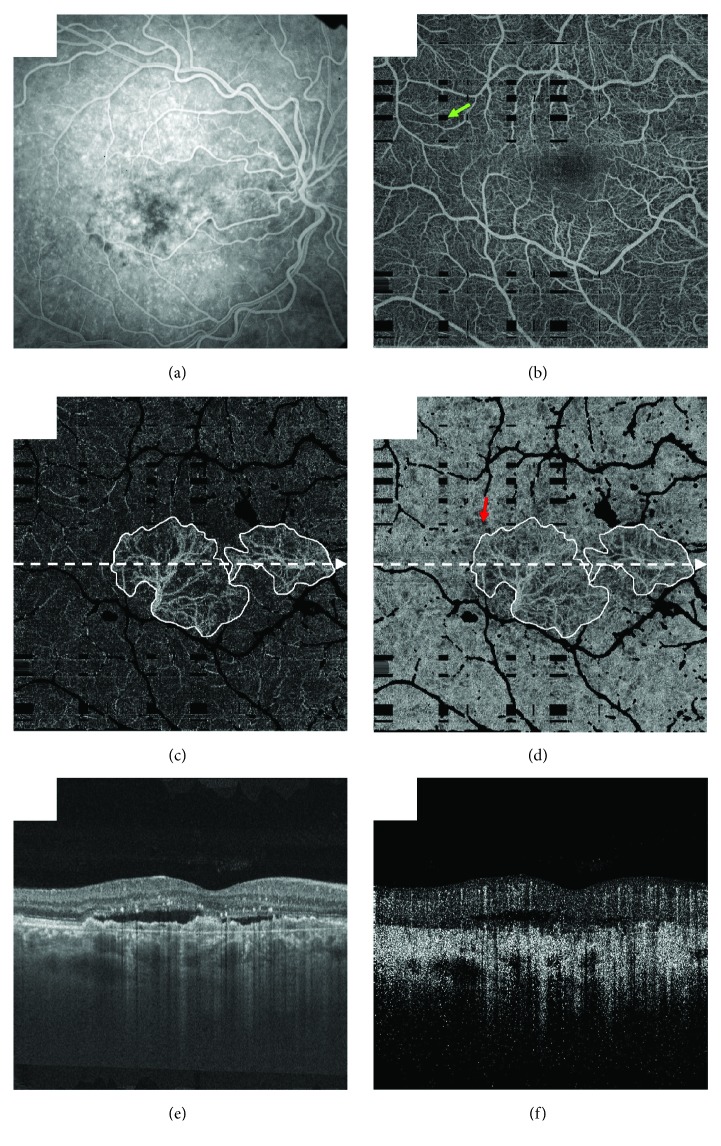
A 65-year-old patient with neovascular age-related macular degeneration (AMD) and treatment-naïve choroidal neovascularization (CNV). (a) Fluorescein angiogram. (b) Projection of the optical coherence tomography (OCT) angiography (OCTA) volume through the depths spanned by the superficial and deep retinal plexuses. The green arrow points to a black rectangular region, which, as a result of patient motion, has absent information (these images were formed by registering and merging orthogonally acquired volumes; at the intersection of motion artifacts in these orthogonal volumes, there is missing information). The field of view is 6 mm × 6 mm. (c) Projection of the OCTA volume through the depths spanned by the CNV lesion; white contours trace the lesion margin. (d) Projection of the OCTA volume from Bruch's membrane to 45 *μ*m below; again, white contours trace the lesion margin, which appears due to projection artifacts. Note that there is choriocapillaris alteration extending beyond the lesion margin (e.g., arrow). (e) OCT B-scan extracted from the position indicated by the dashed white arrows in (c) and (d). (f) OCTA B-scan extracted from the same position. Note that in (b), (c), and (d), projection artifacts from larger overlying retinal vessels have been removed and are shown in black. OCT and OCTA volumes were formed by registering and merging two orthogonally scanned “x-fast” and “y-fast” volumes. Black rectangles in (c) and (d) correspond to intersections of motion in these x-fast and y-fast volumes.

Moult and colleagues studied CNV lesions and the underlying choriocapillaris in patients with neovascular AMD, using an ultrahigh-speed swept-source OCTA. They could visualize 16 of 17 eyes with active CNV, corresponding to 94% sensitivity for CNV detection compared to standard fluorescein angiography. In all these 16 eyes, CNV seemed to originate from regions of severe choriocapillaris alteration. These authors also observed that in 14 of these eyes, CNV lesions were surrounded by a region of severe choriocapillaris alteration (Figure 2) [29]. These findings corroborate what McLeod and colleagues found in their study analyzing postmortem eyes [11].

In 2014, Jia and colleagues analyzed choroidal changes in AMD eyes using OCTA and observed that, in all cases, deep choroidal vessels were easier to detect than in control cases; they hypothesized that this could be caused by loss of choriocapillaris associated with AMD. They also found the absence of choriocapillaris in some areas surrounding CNV lesions [30].

## 6. Conclusion

Significant progress has been made in the understanding of risk factors associated with the development and progression of advanced AMD, either GA or CNV. Nonetheless, the exact underlying mechanisms of tissue damage are still unknown, and the sequence of events involving photoreceptors, RPE, and choriocapillaris loss are still a matter of debate. Pathological changes of the Bruch's membrane, vessel walls, and extracellular deposits must also be considered. In this context, *in vivo* investigation of the choriocapillaris using OCTA may lead to new insights related to underlying disease mechanisms in AMD and may clarify the role of choriocapillaris loss in this vision-threatening disease.
